# Receptor Interaction Profiles of 4-Alkoxy-Substituted 2,5-Dimethoxyphenethylamines and Related Amphetamines

**DOI:** 10.3389/fphar.2019.01423

**Published:** 2019-11-28

**Authors:** Karolina E. Kolaczynska, Dino Luethi, Daniel Trachsel, Marius C. Hoener, Matthias E. Liechti

**Affiliations:** ^1^Division of Clinical Pharmacology and Toxicology, Department of Biomedicine, University Hospital Basel and University of Basel, Basel, Switzerland; ^2^Center for Physiology and Pharmacology, Institute of Pharmacology, Medical University of Vienna, Vienna, Austria; ^3^ReseaChem GmbH, Burgdorf, Switzerland; ^4^Neuroscience Research, pRED, Roche Innovation Center Basel, F. Hoffmann-La Roche Ltd, Basel, Switzerland

**Keywords:** 2,4,5-trimethoxyamphetamine, phenethylamine, 2C-O, 3C-O, receptor, transporter, psychedelic

## Abstract

**Background:** 2,4,5-Trimethoxyamphetamine (TMA-2) is a potent psychedelic compound. Structurally related 4-alkyloxy-substituted 2,5-dimethoxyamphetamines and phenethylamine congeners (2C-O derivatives) have been described but their pharmacology is mostly undefined. Therefore, we examined receptor binding and activation profiles of these derivatives at monoamine receptors and transporters.

**Methods:** Receptor binding affinities were determined at the serotonergic 5-HT_1A_, 5-HT_2A_, and 5-HT_2C_ receptors, trace amine-associated receptor 1 (TAAR1), adrenergic α_1_ and α_2_ receptors, dopaminergic D_2_ receptor, and at monoamine transporters, using target-transfected cells. Additionally, activation of 5-HT_2A_ and 5-HT_2B_ receptors and TAAR1 was determined. Furthermore, we assessed monoamine transporter inhibition.

**Results:** Both the phenethylamine and amphetamine derivatives (*K*_i_ = 8–1700 nM and 61–4400 nM, respectively) bound with moderate to high affinities to the 5-HT_2A_ receptor with preference over the 5-HT_1A_ and 5-HT_2C_ receptors (5-HT_2A_/5-HT_1A_ = 1.4–333 and 5-HT_2A_/5-HT_2C_ = 2.1–14, respectively). Extending the 4-alkoxy-group generally increased binding affinities at 5-HT_2A_ and 5-HT_2C_ receptors but showed mixed effects in terms of activation potency and efficacy at these receptors. Introduction of a terminal fluorine atom into the 4-ethoxy substituent by trend decreased, and with progressive fluorination increased affinities at the 5-HT_2A_ and 5-HT_2C_ receptors. Little or no effect was observed at the 5-HT_1A_ receptor for any of the substances tested (*K*_i_ ≥ 2700 nM). Phenethylamines bound more strongly to the TAAR1 (*K*_i_ = 21–3300 nM) compared with their amphetamine analogs (*K*_i_ = 630–3100 nM).

**Conclusion:** As seen with earlier series investigated, the 4-alkyloxy-substituted 2,5-dimethoxyamphetamines and phenethylamines share some trends with the many other phenethylamine pharmacophore containing compounds, such as when increasing the size of the 4-substituent and increasing the lipophilicity, the affinities at the 5-HT_2A/C_ subtype also increase, and only weak 5-HT_2A/C_ subtype selectivities were achieved. At least from the binding data available (i.e., high affinity binding at the 5-HT_2A_ receptor) one may predict mainly psychedelic-like effects in humans, at least for some of the compound investigated herein.

## Introduction

The serotonin (5-hydroxytryptamine, 5-HT) 5-HT_2_ receptor family is involved in monitoring the balance of several central nervous system processes including sleep, appetite or sexual activity as well as maintaining the regulation of the cardiovascular system. Unsurprisingly, a lack of homeostasis in the 5-HT_2_ receptor mediated processes controlling neurotransmission is thought to play a large role in the occurrence of several mental disorders like anxiety, depression or schizophrenia.

**Figure 1:**
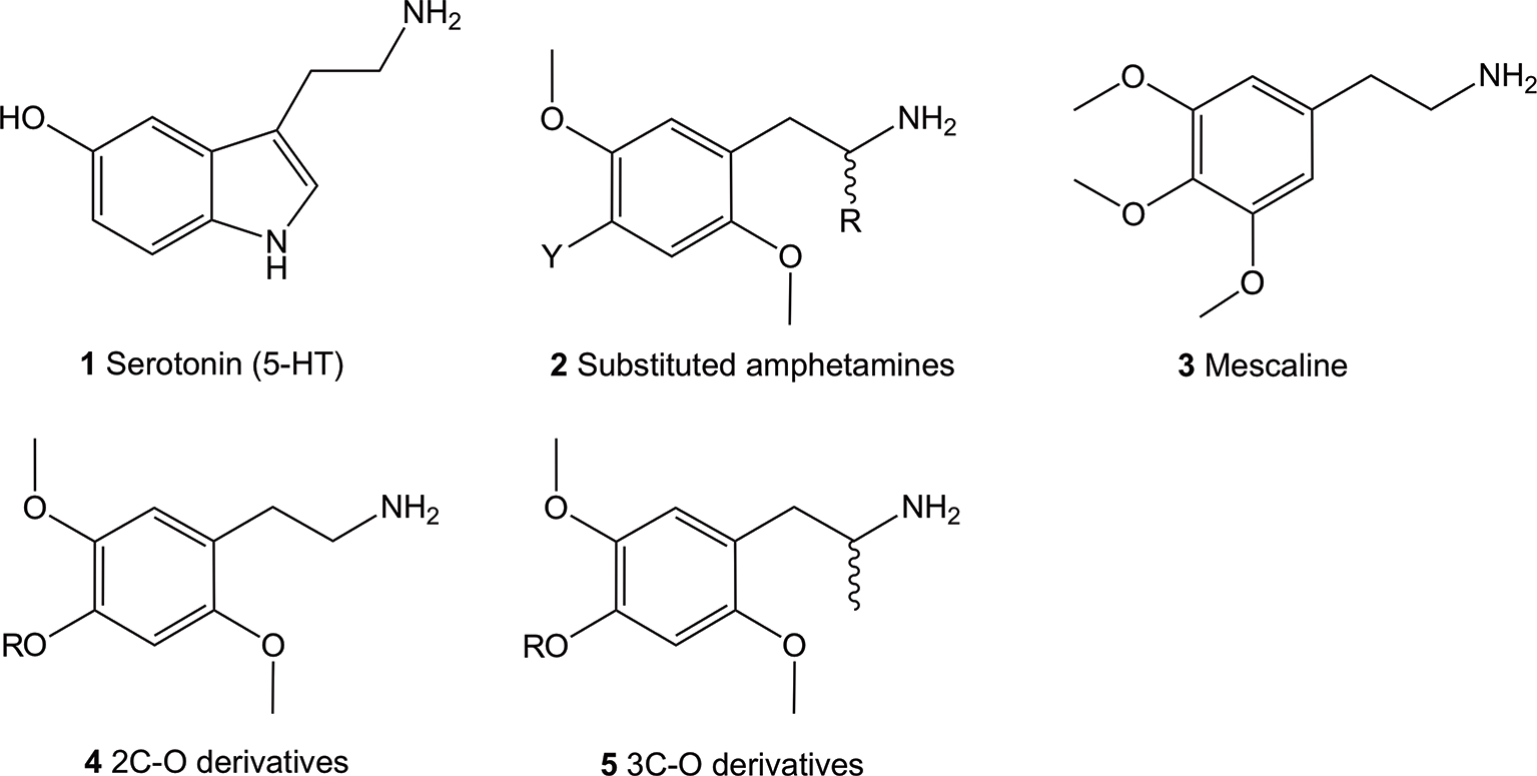
Chemical structures of 5-hydroxytryptamine (5-HT; **1**), 2,5-dimethoxy-4-substituted phenethylamines (**2**), 3,4,5-trimethoxyphenethylamine (mescaline; **3**) and the general structure of the 4-alkoxy-2,5-dimethoxyphenethylamine (**4**) and 4-alkoxy-2,5-dimethoxyamphetamine (**5**) compounds. All amphetamines contain the phenethylamine core.

The 5-HT_2_ receptor family can be subdivided into the 5-HT_2A_, 5-HT_2B_, and 5-HT_2C_ isoforms, all of which are activated by their natural but non-selective, neurotransmitter agonist, 5-HT (**1**) (**Figure 1**; structures **1**-**5**). The lack of selectivity observed for the endogenous neurotransmitter limited its use as a pharmacological tool to characterize the role of each receptor subtype. Investigations using selective ligands associated to selective functional activity are one approach to overcome this issue. Simple aryl-substituted phenethylamines are one example of ligands which lack specific receptor selectivity but show high affinity binding at the 5-HT receptor family (Glennon et al., 1992; Monte et al., 1996; Chambers et al., 2001; Chambers et al., 2002; Whiteside et al., 2002).

The thoroughly investigated 2,5-dimethoxy-4-bromoamphetamine (DOB; **2**, Y = Br, R = Me) (**Figure 1**) binds to the 5-HT_2A_ and 5-HT_2C_ (and 5-HT_2B_) receptors with high affinity but shows relatively low selectivity between the receptor subtypes (Glennon et al., 1992; Monte et al., 1996; Chambers et al., 2001; Chambers et al., 2002; Whiteside et al., 2002). This lack of selectivity is thought to be due to the a high degree of sequence homology between the two receptor subtypes in the ligand binding site located in the transmembrane region (Boess and Martin, 1994).

Up-to-date, hundreds of phenethylamines, mostly synthetic derivatives, are known and have either been investigated for 5-HT_2_ receptor affinities and/or for their psychoactive properties (Aldous et al., 1974; Barfknecht and Nichols, 1975; Nichols et al., 1977; Glennon et al., 1980; Domelsmith et al., 1981; Glennon and Young, 1982; Shulgin and Shulgin, 1991; Glennon et al., 1994; Jacob and Shulgin, 1994; Trachsel, 2003; Trachsel et al., 2013; Rickli et al., 2015; Luethi et al., 2018; Luethi et al., 2019). Many of them have been established as potent psychedelics in man, with mescaline (**3**), the psychedelic ingredient of cacti such as *peyote*, being the first psychedelic phenethylamine synthetically available to man since the year 1919 (Späth, 1919). For this reason, they have been extensively investigated for the past hundred years in animal models and in humans.

The most potent compounds carry the prototypical structure of a phenethylamine with a 2,4,5-subtitution pattern (structure **2**). The 2- and 5- positions are occupied by MeO (methoxy-) groups while the 4-position bares a lipophilic substituent like a halogen, alkyl, alkylsulfanyl or other. Since the introduction of an α-Me (methyl) group (R = Me) onto **2** has a minor influence on affinity binding to the 5-HT_2A/C_ receptors, racemic α-methyl congeners (amphetamines) display about the same affinity at these receptors as their phenethylamine counterparts (Johnson et al., 1990; Glennon et al., 1992; Dowd et al., 2000; Parrish et al., 2005). On the other hand, significant effects of this modification on the dosage and duration of action *in vivo* have been observed in humans (Shulgin and Shulgin, 1991). These are thought to be partially caused by an increased metabolic stability (Glennon et al., 1983; Glennon et al., 1992) and increased hydrophobicity (Nichols et al., 1991). Furthermore, intrinsic activity at the receptor also appear to play a significant role (Nichols et al., 1994), as the α-Me group-containing amphetamines show higher intrinsic activity compared to their phenethylamine counterparts (Parrish et al., 2005).

Overall, a general trend among compounds with the structure **2** which contain small lipophilic substituents (Y = halogen, Me, CF_3_ etc.) on the pivotal 4-position exhibit agonist properties. Conversely, compounds which contain large lipophilic 4-substituents like a longer alkyl chain (such *n*-butyl, 3-phenylpropyl etc.) exhibit antagonist activity (Dowd et al., 2000). Regardless of these observed trends, the transition between these structures is yet to be thoroughly defined.

**Figure 2:**
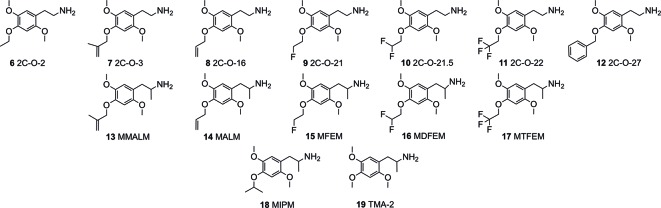
Chemical structures of 4-alkyloxy-2,5-dimethoxyphenethylamines (2C-O derivatives) and 4-alkyloxy-2,5-dimethoxyamphetamines (3C-O) examined in the investigation.

**Figure 3:**
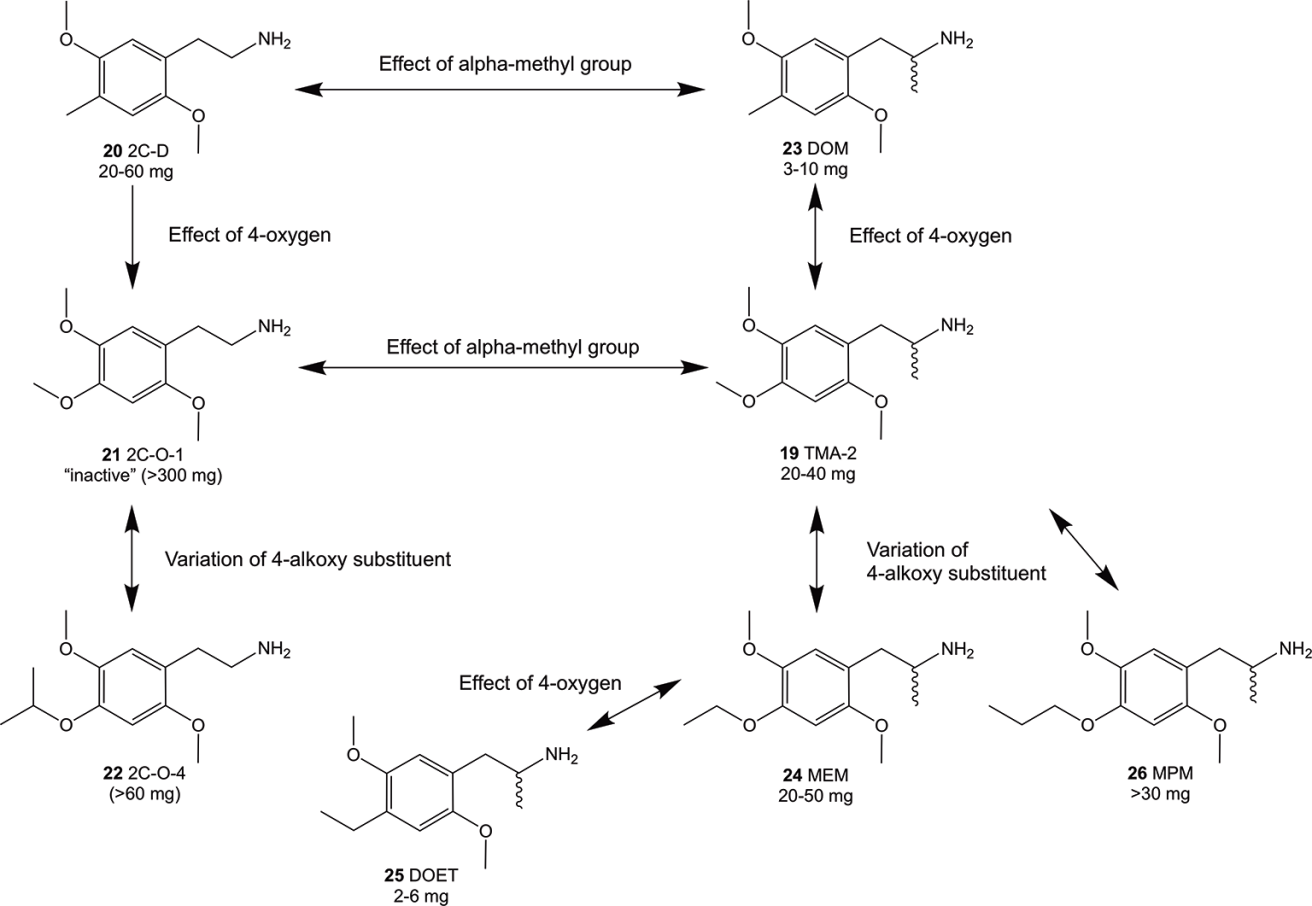
Summary of Shulgin’s early findings on structural modifications and their relation to psychoactive oral doses in humans using phenethylamines and amphetamines related to the 2C-O and 3C-O compounds. Doses were taken from PiHKAL (Shulgin and Shulgin, 1991). In general, an alpha-methylation (compounds **23** and **19**) of the 4-substituted 2,5-dimethoxy compounds increased the psychoactive potency while addition of 4-oxygen decreased potency (compounds **21** and **19**).

In continuation of expanding the data available, we investigated a series of hitherto relatively unknown 4-substituted 2,5-dimethoxyphenethylamines and some of their corresponding α-Me congeners the 4-alkoxy derivatives 2C-O (structures **6-12**) and 3C-O (structures **13-19**) compounds (**Figure 2**). Within these compounds, 2,4,5-trimethoxyamphetamine (TMA-2; **19**, **Figures 2** and **3**) can be considered as the archetypical representative. It is a synthetic compound that induces psychedelic effects in users likely *via* agonistic binding to the serotonergic 5-HT_2A_ receptor, similar to other classic psychedelics (Shulgin and Shulgin, 1991; Vollenweider et al., 1998; Preller et al., 2017). Back in 1966, varying the 3,4,5-trimethoxy substitution pattern to all possible position isomers revealed the importance of a 2,4,5-trimethoxy arrangement in aryl-substituted amphetamines and phenethylamines (Shulgin, 1966). Unlike classical amphetamines, **19** is characterized as a psychedelic with minor stimulant properties and is roughly 6 times more potent in humans than its structural and psychedelic isomer 3,4,5-trimethoxyamphetamine (TMA, structure not shown) (Shulgin and Shulgin, 1991; EMCDDA, 2004).

Since the 1960s, only a handful 4-alkoxy analogs of **19** have been synthesized and described (Shulgin and Shulgin, 1991; Trachsel, 2012). In accordance to the nomenclature introduced by the chemist Alexander Shulgin, they can generally be named as 2C-O derivatives (4-alkoxy-2,5-dimethoxyphenethylamines) and as 3C-O derivatives (4-alkoxy-2,5-dimethoxyamphetamines) (Shulgin and Shulgin, 1991) (structures **4** and **5**, **Figure 1**). The subsequent number is random and has been assigned in the order of planning the chemical synthesis of the compounds. However, as for the 3C-O derivatives naming is following a somewhat different approach: the initials for the three substituents in the 2,4,5-positions are used to build the name. For example, the name MMALM (compound **13**, **Figure 2**) derives from *m*ethoxy *m*eth*a*llyl *m*ethoxy (Shulgin and Shulgin, 1991). Up-to-date, the effects of only few of the 2C-O and 3C-O derivatives have been described chemically and (psycho)pharmacologically (Shulgin and Shulgin, 1991; Trachsel et al., 2013). Although the 2C-O derivatives initially examined by Shulgin were shown to be fairly inactive in humans (2C-O-1; **21** and 2C-O-4; **22**, **Figure 3**) some derivatives such as **19** and 2,5-dimethoxy-4-ethoxyamphetamie (MEM) (**24**) displayed psychedelic activity (**Figure 3**) (Shulgin and Shulgin, 1991). However, upon further increasing chain length to a 4-propyloxy (MPM; **26**) or 4-butyloxy (MBM; structure not shown) substituent, again no psychoactive effects could be observed on comparable doses as used for **19** and **24**. The rather mixed results of low human potency and inactivity was one of the reasons Shulgin did not further evaluate the structure-activity relationship (SAR) of the 2C-O and 3C-O derivatives. Up-to-date, it remains unclear whether the early observations are due to pharmacokinetic properties such as a difference in metabolism or pharmacodynamic properties like differences in 5-HT receptor target interaction potency. Thus, in order to get more insight into SAR, a series of new 2C-O and 3C-O analogs (**6-19**, **Figure 2**) with potential to induce psychedelic properties (Trachsel, 2012) was prepared and pharmacologically investigated herein.

In the current study, we investigated the receptor binding and activation properties of several 2C-O derivatives and some of their 3C-O counterparts (**Figure 2**) at serotonergic, dopaminergic, and adrenergic receptors and trace-amine associated receptor 1 (TAAR1). The modifications found on these derivatives at the 4-position included introduction of one or more fluorines (mono-/di-/tri-fluorination at the terminal H_3_C-group of the 4-ethoxy substituent) or extending of the 4-alkoxy-group by homologation, alkenylation and/or branching. Furthermore, we investigated the potential of these derivatives to bind and inhibit norepinephrine, dopamine, and 5-HT transporters (NET, DAT, and SERT, respectively) in human transporter-transfected human embryonic kidney (HEK) 293 cells.

## Materials and Methods

### Drugs

All of the 2,5-dimethoxy-4-substituted phenethylamines (2C-O-2, 2C-O-16, 2C-O-3, 2C-O-27, 2C-O-21, 2C-O-21.5 and 2C-O-22) and the 2,5-dimethoxy-4-substituted amphetamines (TMA-2, 2,5-dimethoxy-4-isopropoxyamphetamine (MIPM), 4-allyloxy-2,5-dimethoxyamphetamine (MALM), 2,5-dimethoxy-4-methallyloxyamphetamine (MMALM), 2,5-dimethoxy-4-(2-fluoroethoxy)amphetamine (MFEM), 4-(2,2-difluoroethoxy)-2,5-dimethoxyamphetamine (MDFEM), and 2,5-dimethoxy-4-(2,2,2-trifluoroethoxy)amphetamine (MTFEM). all racemates) were synthetized in the hydrochloride form by ReseaChem (Burgdorf, Switzerland) as previously described (Shulgin and Shulgin, 1991; Trachsel, 2003; Trachsel et al., 2013). Purity for all described substances was >98%. [^3^H]5-HT (80.0 Ci/mmol) was acquired from Anawa (Zurich, Switzerland). [^3^H]dopamine (30.0 Ci/mmol) and [^3^H]norepinephrine (13.1 Ci/mmol) were purchased from Perkin-Elmer (Schwerzenbach, Switzerland).

### Radioligand Receptor and Transporter Binding Assays

The radioligand receptor and transporter binding assays were performed according to methods previously described in detail (Luethi et al., 2018). In summary, cell membrane preparations overexpressing respective receptors (human genes, except for rat and mouse genes for TAAR1) or transporters (human genes) were briefly incubated with radiolabeled selective ligands at a concentration equal to the dissociation constant (*K*_d_). The cell membrane preparations were obtained from various cell lines including a Chinese hamster ovary cell line (α_1A_ adrenergic receptor), Chinese hamster lung cell line (α_2A_ adrenergic receptor) and HEK 293 cell line (5-HT_1A_, 5-HT_2A_, 5-HT_2C_, TAAR1, D_2_, NET, DAT, SERT). Ligand displacement by the substances was measured. Specific binding of the radioligand to the target site was defined by measuring the difference between total binding and nonspecific binding (calculated in the presence of the respective receptor competitor in excess).

The following radioligands and the respective competitors were used: 0.90 nM [^3^H]8-hydroxy-2-(dipropylamino)tetralin (8-OH-DPAT) and 10 µM pindolol (5-HT_1A_ receptor), 0.40 nM [^3^H]ketanserin and 10 µM spiperone (5-HT_2A_ receptor), 1.4 nM [^3^H]mesulgerine, and 10 µM mianserin (5-HT_2C_ receptor), 3.5 nM or 2.4 nM (rat and mouse TAAR1, respectively) [^3^H]RO5166017 and 10 µM RO5166017 (rat and mouse TAAR1), 0.11 nM [^3^H]prazosin and 10 µM chlorpromazine (α_1_ adrenergic receptor), 2 nM [^3^H]rauwolscine and 10 µM phentolamine (α_2_ adrenergic receptor), 1.2 nM [^3^H]-spiperone and 10 µM spiperone (dopaminergic D_2_ receptor), 2.9 nM N-methyl-[^3^H]-nisoxetine and 10 µM indatraline (NET), 1.5 nM [^3^H]citalopram, and 10 µM indatraline (SERT), 3.3 nM [^3^H]WIN35,428 and 10 µM indatraline (DAT).

### Activity At the Serotonin 5-HT_2A_ Receptor

Activity at the 5-HT_2A_ receptor was examined as previously described (Luethi et al., 2018). In summary, mouse embryonic fibroblasts (NIH-3T3 cells) expressing the human 5-HT_2A_ receptor were seeded at a density of 70,000 cells per 100 µl in poly-D-lysine-coated 96-well plates. The cells were then incubated in HEPES-Hank’s Balanced Salt Solution (HBSS) buffer (Gibco) for 1 h at 37°C. Next, the plates were incubated in 100 µl (per well) dye solution for 1 h at 37°C (fluorescence imaging plate reader [FLIPR] calcium 5 assay kit; Molecular Devices, Sunnyvale, CA, USA). Once inside the FLIPR, the plates were exposed to 25 µl of test drugs which were diluted in HEPES-HBSS buffer composed of 250 mM probenecid while online. Using nonlinear regression, the rise in fluorescence was measured and EC_50_ values were calculated from the concentration-response curves. The efficacy was calculated relative to 5-HT activity which was defined as 100%. This assay was mainly used to determine whether the compounds were active, while 5-HT_2A_ receptor binding is considered to be more relevant to predict hallucinogenic potency in humans (Luethi and Liechti, 2018).

### Activity At the Serotonin 5-HT_2B_ Receptor

Activity at the 5-HT_2B_ receptor was examined as previously described (Luethi et al., 2018). In summary, HEK 293 cells expressing the human 5-HT_2B_ receptor were seeded at a density of 50,000 cells per well in 96-well poly-D-lysine-coated plates overnight at 37°C. The cells were then incubated in growth medium consisting of high glucose Dulbecco’s modified Eagle’s medium (DMEM) (Invitrogen, Zug, Switzerland), 10% fetal calf serum (non-dialyzed, heat-inactivated), 250 mg/l Geneticin and 10 ml/l PenStrep (Gibco), overnight at 37°C. After removal of the growth medium *via* snap inversion, calcium indicator Fluo-4-solution (100 µl) was injected into each well (Molecular Probes, Eugene, OR, USA) and incubated for 45 min at 31°C. Afterwards, the Fluo-4 solution was removed (snap inversion) and a further 100 µl of the Fluo-4 solution was added and incubated (45 min, 31°C). Thereafter, using the EMBLA cell washer, the cells were washed just before testing with HBSS and 20 mM HEPES and exposed to 100 µl of assay buffer. The well plate was positioned in the FLIPR and while online, 25 µl of the test compounds diluted in assay buffer were added. Concentration-response curves were calculated using nonlinear regression, and EC_50_ values were obtained. The maximal activity at the receptors was calculated relative to 5-HT activity which was defined as 100%. Since setting up a stable and reliable binding assay for the 5-HT_2B_ receptor has been proven to be difficult in the past, we did not try to include binding data for this receptor in our investigation. However, because the 5-HT_2B_ receptor activity is important for determining the potential cardiotoxicity of a derivative, we have included this data to estimate whether any substances have the potential to induced endocardial fibrosis.

### Activity At the Human TAAR1

Activity at the human TAAR1 was examined as previously described in full detail (Luethi et al., 2018). In summary, human TAAR1 expressing recombinant HEK 293 cells were grown in 250 ml falcon culture flasks containing 30 ml of high glucose DMEM (10% heat inactivated fetal calf serum, 500 µg/ml Geneticin [Gibco, Zug, Switzerland] and 500 µg/ml hygromycin B) at 37°C and 5% CO_2_/95% air. At 80-90% confluency, the cells were collected. The medium was aspirated, cells were washed with phosphate-buffered saline (PBS) and then trypsinized for 5 min at 37°C with 5 ml of trypsin/EDTA solution. Next, 45 ml of medium was added and the mixture was transferred into a falcon tube. After centrifugation (900 rpm, 3 min, RT), the supernatant was aspirated and the remaining cell pellet was resuspended in fresh medium to 5 × 10^5^ cells/ml. Using a multipipette, 100 µl of cells were transferred (80,000 cells/well) into a 96-well plate (BIOCOAT 6640, Becton Dickinson, Allschwil, Switzerland) and incubated for 20 h at 37°C. For the cAMP assay, the medium was aspirated replaced with 50 µL PBS without Ca^2+^ and Mg^2+^ ions. The PBS was then extracted using snap inversion and the plate was softly tapped against tissue; 90 µL of Krebs-Ringer Bicarbonate buffer (Sigma-Aldrich) containing 1 mM IBMX was added and incubated for 60 min at 37°C and 5% CO_2_/95% air. A concentration range between 300 pM and 30 µM of test compounds was examined in duplicate. A standard curve with a range of cAMP concentrations (0.13 nM to 10 µM) was created per 96-well plate. Each experiment was accompanied with a reference plate that included three compounds; RO5256390, β-phenylethylamine, and p-tyramine. The cells were exposed to either 30 µl of compound solution, 30 µl of β-phenylethylamine (as maximal response), or a basal control in PBS (containing 1 mM IBMX) for 40 min at 37°C. Next, under forceful shaking using black lids, the cells were exposed to 50 µl of 3x detection mix solution (composed of Ru-cAMP Alexa700 anti-cAMP antibody and lysis buffer) for 120 min at room temperature in order to lyse the cells. Using the NanoScan reader (Innovative Optische Messtechnik, Berlin, Germany; 456 nm excitation wavelength; 630 and 700 nm emission wavelengths), the fluorescence was examined and the fluorescence resonance energy transfer (FRET) signal was determined using the following equation; FRET (700 nm) − P × FRET (630 nm), where P = Ru (700 nm)/Ru (630 nm). Receptor binding affinity at the human TAAR1 was not determined since unfortunately, there are no suitable radioligands available for this receptor.

Functional activity of the derivatives was only examined at the human TAAR1 and not at the mouse and rat TAAR1, because functional assays are not set up.

### Monoamine Uptake Transporter Inhibition

The monoamine uptake transporter inhibition for 2,5-dimethoxy-4-alkyloxy phenethylamines and amphetamines was examined in HEK 293 cells stably transfected with the human 5-HT, norepinephrine and dopamine transporters (hSERT, hNET, hDAT) as previously described (Luethi et al., 2018). Only one single high concentration was tested to exclude activity at the transporters at pharmacologically relevant concentrations. Briefly, the cells were cultured in DMEM (Gibco, Life Technologies, Zug, Switzerland) containing 10% fetal bovine serum (Gibco) and 250 µg/ml Geneticin (Gibco). At 70-90% confluency, the cells were detached and resuspended in Krebs-Ringer Bicarbonate Buffer (Sigma-Aldrich, Buchs, Switzerland) at a density of 3 × 10^6^ cells per ml of buffer. For [^3^H]dopamine uptake experiments, the buffer additionally contained 0.2 mg/ml of ascorbic acid. 100 µL of cell suspension was added to each well into a round bottom 96-well plate. The cells were then incubated with 25 µL buffer containing the test drug (10 µM), vehicle control (0.1% dimethyl sulfoxide) or transporter-specific inhibitors (10 µM fluoxetine (SERT), 10 µM mazindol (DAT) or 10 µM nisoxetine (NET)) for 10 min by shaking on a rotary shaker (450 rpm) at room temperature. Uptake transport was initiated by adding [^3^H]5-HT, [^3^H]dopamine, or [^3^H]norepinephrine at a final concentration of 5 nM to the mixture. After 10 min, 100 µL of the cell mixture was transferred to 500 µL microcentrifuge tubes containing 50 µL of 3 M KOH and 200 µL silicon oil (1:1 mixture of silicon oil types AR 20 and 200; Sigma-Aldrich). The tubes were centrifuged for 3 min at 16,550 g, to allow the transport of the cells through the silicon oil layer into the KOH layer. The tubes were frozen in liquid nitrogen and the cell pellet was cut into 6 ml scintillation vials (Perkin-Elmer) containing 0.5 ml lysis buffer (1% NP-40, 50 mM NaCl, 0.05 M TRIS-HCl, 5 mM EDTA, and deionized water). The samples were shaken for 1 h before 3 ml of scintillation fluid (Ultimagold, Perkin Elmer, Schwerzenbach, Switzerland) was added. Monoamine uptake was then quantified by liquid scintillation counting on a Packard Tri-Carb Liquid Scintillation Counter 1900 TR. Uptake in the presence of the selective inhibitors was determined to be nonspecific and subtracted from the total counts.

### Statistical Analysis

Calculations were performed using Prism 7.0a software (GraphPad, San Diego, CA, USA). IC_50_ values of radioligand binding were determined by calculating nonlinear regression curves for a one-site model using at least three independent 10-point concentration-response curves for each substance. The *K*_i_ values correspond to the dissociative constant for the inhibitor and were calculated using the Cheng-Prusoff equation. Nonlinear regression concentration-response curves were used to determine EC_50_ values for 5-HT_2A_ and 5-HT_2B_ receptor activation. Maximal activation activity (efficacy) is expressed relative to the activity of 5-HT, which was used as a control and set to 100%. Monoamine uptake of three to four independent experiments was compared to control using 1-way analysis of variance followed by a Dunett’s multiple-comparison test. Monoamine uptake of 3,4-methylenedioxymethamphetamine (MDMA) was included as comparison. Receptor affinity binding (*K*_i_) <50 nM was defined as high affinity binding, <500 nM moderate affinity binding while *K*_i_ >1000 nM was defined as low affinity binding. Activation efficacy (max %) < 85% was defined as partial agonism while max % > 85% was defined as full agonism.

## Results

### Binding and Activation At 5-HT Receptors

5-HT receptor binding affinities and activation potencies are listed in **Table 1**. The well-explored phenethylamine 2C-B (Kurrasch-Orbaugh et al., 2003; Rickli et al., 2015; Papaseit et al., 2018) was included for comparison. All of the 2C-O derivatives (**Figure 2**, structures **6–12**) exhibited only very weak binding properties to the 5-HT_1A_ receptor (*K*_i_ = 2.7–5.5 µM) and the 2,5-dimethoxy-4-alkyloxy substituted amphetamines (**Figure 2**, structures **13–19**) did not bind to the receptor (*K*_i_ > 5600 nM).

**Table 1:** Serotonin receptor binding affinities and activation potencies of 4-alkoxy-substituted 2,5-dimethoxyphenethylamines and amphetamines.

		h5-HT_1A_	h5-HT_2A_			h5-HT_2B_		h5-HT_2C_	Selectivity (binding ratios)	
		Receptor binding *K*_i_ ± SD [nM] [^3^H]8-OH-DPAT	Receptor binding *K*_i_ ± SD [nM] [^3^H]ketanserin	Activation potency EC_50_ ± SD [nM]	Activation efficacy max ± SD [%]	Activation potency EC_50_ ± SD [nM]	Activation efficacy max ± SD [%]	Receptor binding *K*_i_ ± SD [nM] [^3^H]mesulgerine	5-HT_2A_/5-HT_1A_	5-HT_2A_/5-HT_2C_
**4-alkoxy-substituted 2,5-dimethoxyphenethylamines**										
**6**	2C-O-2	3600 ± 400	670 ± 90	16 ± 3	50 ± 10	49 ± 44	85 ± 17	2300 ± 1200	5.4	3.4
**7**	2C-O-3	2700 ± 200	40 ± 8	0.5 ± 0.3	70 ± 4	68 ± 21	50 ± 22	150 ± 30	68	3.8
**8**	2C-O-16	4500 ± 500	140 ± 40	4.9 ± 2.5	84 ± 4	120 ± 70	42 ± 13	470 ± 40	32	3.4
**9**	2C-O-21	5500 ± 0	1700 ± 600	53 ± 15	44 ± 17	900 ± 450	39 ± 26	3600 ± 1900	3.2	2.1
**10**	2C-O-21.5	2900 ± 200	1000 ± 100	2600 ± 1600	30 ± 8	480 ± 260	28 ± 23	4200 ± 1500	2.9	4.2
**11**	2C-O-22	3300 ± 200	440 ± 60	460 ± 140	35 ± 8	250 ± 10	28 ± 20	1900 ± 400	7.5	4.3
**12**	2C-O-27	2700 ± 100	8.1 ± 1	76 ± 80	36 ± 16	480 ± 190	39 ± 16	110 ± 50	333	14
**4-alkoxy-substituted 2,5-dimethoxyamphetamines**										
**13**	MMALM	NA	61 ± 0	1.5 ± 0.1	95 ± 4	29 ± 13	90 ± 13	290 ± 110	>92	4.8
**14**	MALM	>5600	150 ± 0	2.9 ± 0.5	89 ± 10	9.5 ± 3.3	101 ± 6	900 ± 220	>37	6
**15**	MFEM	>5600	1900 ± 900	330 ± 180	56 ± 36	270 ± 210	41 ± 22	11000 ± 3000	>2.9	5.8
**16**	MDFEM	>5600	980 ± 620	140 ± 80	48 ± 23	130 ± 80	21 ± 8	6700 ± 2400	>5.7	6.8
**17**	MTFEM	>5600	460 ± 240	19 ± 1	80 ± 6	200 ± 30	4.8 ± 3.5	2400 ± 800	>12	5.2
**18**	MIPM	>5600	4400 ± 2100	990 ± 330	47 ± 13	180 ± 120	20 ±14	9030 ± 2390	>1.4	2.1
**19**	TMA-2	>17000	1300 ± 700	190 ± 90	84 ± 10	270 ± 0	78 ± 5	5300 ± 1600	>13	4.1
**Reference substance**										
	2C-B^a^	311±46	6.9 ± 1.8	2.1 ± 0.8	92 ± 8	75 ± 14	52 ± 26	43 ± 4	45	6.2

K_i_ and EC_50_ values are given as nM (mean ± SD); activation efficacy (E_max_) is given as percentage of maximum ± SD. NA, not assessed.

a Data previously published in (Luethi et al., 2018).

The 2C-O derivatives bound to the 5-HT_2A_ receptor with submicromolar affinity (*K*_i_ = 8–1000 nM), with the exception of 2C-O-21 (**9**) (*K*_i_ = 1700 nM). Additionally, the 2C-O derivatives were partial agonists with EC_50_ values in the ranges of 16–2600 nM and activation efficacies of 30–84%. Some of the 3C-O derivatives, the 4-alkoxy-2,5-dimethoxy substituted amphetamines bound to the 5-HT_2A_ receptor with submicromolar affinity (*K*_i_ = 61–980 nM), with the exception of MFEM (**15**), MIPM (**18**), and TMA-2 (**19**) (*K*_i_ = 1300–4400 nM). The 3C-O compounds activated the receptor with EC_50_ values in the ranges of 2–990 nM. Compounds MALM (**14**) and MMALM (**13**) proved to be full agonists with activation efficacies of 89% and 95%, respectively. The remaining compounds activated the receptor as partial agonists with activation efficacies in the range of 47–84%.

All compounds activated the 5-HT_2B_ receptor at submicromolar concentrations. Compounds 2C-O-2 (**6**), MALM (**14**), and MMALM (**13**) were full agonists with activation efficacies of 85–101%. The remaining compounds were partial agonists (activation efficacies in the range of 20–78%) with the exception of MTFEM (**17**) which had only negligible activation efficacy (5%).

Compounds 2C-O-3 (**7**), 2C-O-16 (**8**), 2C-O-27 (**12**), MALM (**14**), and MMALM (**13**) potently bound to the 5-HT_2C_ receptor (*K*_i_ = 15–900 nM). The remaining compounds bound with affinities in the range of 1900–11000 nM.

### Interactions With Non-Serotonergic Monoamine Receptors and Transporters

The non-serotonergic monoamine receptors and transporters binding affinities are listed in **Table 2** with MDMA as reference for comparison. The 2C-O derivatives did not activate the human TAAR1 except for 2C-O-2 (**6**) and 2C-O-22 (**11**), which activated the receptor in the micromolar range (EC_50_ = 3600–9600 nM). The 4-alkyloxy-2,5-dimethoxy substituted amphetamines did not activate the human TAAR1.

**Table 2:** Monoamine receptor and transporter binding affinities of 4-alkoxy-subsituted 2,5-dimethoxyphenethylamines and amphetamines.

		human TAAR1	rat TAAR1	mouse TAAR1	α_1A_	α_2A_	D_2_	hNET	hDAT	hSERT
		EC_50_ ± SD [nM]	*K*_i_ ± SD [nM] [^3^H] RO5166017	*K*_i_ ± SD [nM] [^3^H] RO5166017	*K*_i_ ± SD [nM] [^3^H] prazosin	*K*_i_ ± SD [nM] [^3^H] rauwolscine	*K*_i_ ± SD [nM] [^3^H] spiperone	*K*_i_ ± SD [nM] N-methyl-[^3^H]nisoxetine	*K*_i_ ± SD [nM] [^3^H] WIN35,428	*K*_i_ ± SD [nM] [^3^H] citalopram
**4-alkoxy-substituted 2,5-dimethoxyphenethylamines**										
**6**	2C-O-2	9600 ± 6340	340 ± 70	4000 ± 1100	>6500	2600 ± 100	>4800	>9700	>8700	>8600
**7**	2C-O-3	> 30000	130 ± 0	1100 ± 400	>6500	180 ± 10	>4800	>9700	>8700	>8600
**8**	2C-O-16	> 30000	260 ± 70	2500 ± 1000	>6500	620 ± 20	>4800	>9700	>8700	>8600
**9**	2C-O-21	> 30000	410 ± 40	> 4900	>6500	3600 ± 500	>4800	>9700	>8700	>8600
**10**	2C-O-21.5	> 30000	250 ± 20	> 4800	>6500	1900 ± 100	>4800	>9700	>8700	>8600
**11**	2C-O-22	3600 ± 2400	240 ± 60	2500 ± 400	>6500	1800 ± 100	>4800	>9700	>8700	>8600
**12**	2C-O-27	> 30000	21 ± 3	650 ± 190	>6500	570 ± 30	>4800	>9700	6100 ± 400	>8600
**4-alkoxy-substituted 2,5-dimethoxyamphetamines**										
**13**	MMALM	> 30000	630 ± 150	1700 ± 900	>6500	2400 ± 200	>4800	>9700	>8700	>8600
**14**	MALM	> 30000	1100 ± 200	2000 ± 400	>6500	>4800	>4400	>9700	>8700	>8600
**15**	MFEM	> 30000	1640 ± 300	> 4900	>6500	>4800	>4800	>9700	>8700	>8600
**16**	MDFEM	> 30000	1200 ± 200	> 4800	>6500	>4800	>4800	>9700	>8700	>8600
**17**	MTFEM	> 30000	1300 ± 100	4500 ± 500	>6500	>4800	>4800	>9700	>8700	>8600
**18**	MIPM	> 30000	2900 ± 700	> 4800	>6500	>4800	>4800	>9700	>8700	>8600
**19**	TMA-2	NA	3100 ± 100	> 4400	>6500	>4700	>13000	>8700	>8500	>7500
**Reference substance**										
	MDMA^a^	NA	370 ± 120	2400 ± 1100	>6000	15000 ± 200	25200 ± 2000	30500 ± 8000	6500 ± 2500	13300 ± 600

K_i_ and EC_50_ values are given as nM (mean ± SD); activation efficacy (E_max_) is given as percentage of maximum ± SD. NA, not assessed.

a Data previously published in (Simmler et al., 2013).

All 2C-O derivatives showed moderate to relatively high affinities to the rat TAAR1 (*K*_i_ = 21–410 nM). In contrast, the 3C-O derivatives generally only weakly bound to the rat TAAR1 (*K*_i_ = 1100–3100 nM), with exception for MMALM (**13**) (*K*_i_ = 630 nM), which showed a modest affinity. The 2C-O derivatives also weakly bound to the mouse TAAR1 (*K*_i_ = 650–4000 nM) with 2C-O-27 (**12**) being the most potent compound. Compounds 2C-O-21 (**9**) and 2C-O-21.5 (**10**) did not bind to mouse TAAR1 in the examined concentration range (*K*_i_ > 4800 nM). The amphetamine derivatives did not bind to the mouse TAAR1 in the examined concentration range (*K*_i_ > 4400 nM), except for MMALM (**13**), MALM (**14**) and MTFEM (**17**) which showed weak binding properties (*K*_i_ = 1700–4500 nM).

None of the phenethylamines or amphetamines bound to the adrenergic α_1A_ (*K*_i_ > 6500 nM) or dopaminergic D_2_ receptors (*K*_i_ > 4400 nM) in the examined concentration range. Compounds 2C-O-3 (**7**), 2C-O-16 (**8**), and 2C-O-27 (**12**) bound to the α_2A_ receptor in the submicromolar range (*K*_i_ = 180–620 nM); the other 2C-O derivatives bound only weakly to the α_2A_ receptor (*K*_i_ = 1800–3600 nM). With exception for MMALM (**13**) (*K*_i_ = 2400 nM), none of the 3C-O derivatives bound to the α_2A_ receptor in the examined concentration range (*K*_i_ > 4700 nM). Among the tested 5-HT, dopamine and norepinephrine transporter interactions, **12** proved to be the only compound able to bind to the DAT (*K*_i_ = 6.1 µM), none of the remaining 2C-O and 3C-O compounds bound to the monoamine transporters at investigated concentrations.

### Monoamine Transporter Inhibition

None of the investigated compounds significantly inhibited monoamine uptake transporters (IC_50_ > 10 µM) (**Supplementary Figure 1**).

## Discussion

### Serotonin 5-HT_2A_ *vs.* 5-HT_2C_ Receptor Binding and Subtype Selectivity

Despite the use of a limited set of compounds, some trends were observed. We found that 2,5-dimethoxy-4-alkyloxy substituted phenethylamine derivatives (**Figure 2**, structures **6-12**) showed binding preference at the 5-HT_2A_ over the 5-HT_2C_ receptor and bound to the 5-HT_1A_ receptor. The corresponding 3C-O derivatives (**Figure 2**, structures **13-19**) displayed a comparable 5-HT_2A_ *vs.* 5-HT_2C_ binding preference but did not bind to the 5-HT_1A_ receptor (section 4.2). The observed moderate 5-HT_2A_ *vs.* 5-HT_2C_ subtype selectivity is in accordance with the many other phenethylamine and amphetamine ligands described by others (Glennon et al., 1980; Glennon et al., 1986; Glennon et al., 1994; Nichols et al., 1994; Nelson et al., 1999; Nichols et al., 2015; Rickli et al., 2015; Nichols, 2016; Rickli et al., 2016; Luethi et al., 2018).

The extension of the 4-alkoxy group was found to increase the binding affinity at the 5-HT_2A_ and 5-HT_2C_ receptors for both phenethylamine and amphetamine derivatives, a trend also in accordance with previous studies on extending a lipophilic 4-substituent in 2,5-dimethoxyphenethylamines (Dowd et al., 2000; Luethi et al., 2018). For 2C-O-16 (**8**; 4-allyloxy) and 2C-O-3 (**7**; 4-methallyloxy) it remains unclear whether the increased affinities at the 5-HT_2A_ and 5-HT_2C_ receptors originate from increasing substituent size solely or whether oxidation to their alkenyl pharmacopore contributes to this as well. Generally, an increased lipophilicity also increases affinities to these receptors and thus both effects may be contributory. Introduction of one (2C-O-21; **9**) or two fluorines (2C-O-21.5; **10**) onto the terminal carbon atom of the 4-ethoxy group lead to decreasing affinities at both 5-HT_2A_ and 5-HT_2C_ receptors compared to 2C-O-2 (**6**). In comparison, 2C-O-22 (**11**) bearing a 4-trifluoroethoxy substituent showed slightly increased affinity over the non-fluorinated compound **6** at the 5-HT_2A_ and 5-HT_2C_ receptors. However, when considering the fluorinated compounds **9**-**11** solely, progressive fluorination leads to increased affinities.

A similar trend was observed for the 4-alkoxy substituted 2,5-dimethoxyamphetamines. Increasing the 4-alkoxy substituent lead to increased affinities, with MIPM (**18**) being an exception: although its 4-isopropyloxy substituent may be considered to be more lipophilic than an ethoxy group, it may have some unfavorable steric bulkiness in that binding area. Even though a benzyloxy substituent is even bulkier (and leads to the highest affinities among these and other compounds at this receptor) (Luethi et al., 2018), the isopropyl group is branched directly at its carbon bound to the oxygen. This potentially may force the 5-MeO group more prominently to an out-of-plane orientation leading to decreased 5-HT_2A_ and 5-HT_2C_ receptor interactions (Monte et al., 1996). Yet, within the 4-alkylthio substituted 2,5-dimethoxyphenethylamines (2C-T and ALEPH derivatives; structures not shown), a 4-isopropylthio substituent proved to be highly efficient in causing psychedelic effects in man, while its affinities where distinctly lower than e.g. those of the corresponding 4-methallylthio or 4-propylthio derivatives, which are both active in man at similar doses (Shulgin and Shulgin, 1991; Luethi et al., 2018). Clearly, other factors, such as lipophilicity, receptor activation, functional selectivity, and monoamine oxidase (MAO) and cytochrome P450 (CYP) metabolism, may influence the dose and effects in man. Also, branching the alkyl chain geminally to the attached oxygen may be more detrimental than when attached to a sulphur atom in respect to 5-HT_2A/C_ affinities, considering the former to have a significantly lower steric bulkiness. Being branched “closer” to the aromatic nucleus due to the smaller oxygen atom, the negligibly active or even psychedelically inactive 2C-O-4 (**22**) (Shulgin and Shulgin, 1991) may also be affected by these steric effects.

Increasing fluorination of the terminal carbon in the 4-ethoxy substituent of the 3C-O derivatives investigated lead to increased affinities at the 5-HT_2A_ and 5-HT_2C_ receptor subtypes (the fluorine-free counterpart MEM; **24** (Shulgin and Shulgin, 1991; Halberstadt et al., 2019) was not available for this study). Additionally, extension of the 4-alkoxy group and increasing the number of fluoro substituents in 3C-O derivatives increased the binding selectivity for 5-HT_2A_ over 5-HT_1A_ receptors.

It is well known that psychedelic phenethylamines and amphetamines bind to both 5-HT_2C_ and 5-HT_2A_ receptors (Kurrasch-Orbaugh et al., 2003; Nichols, 2004; Moya et al., 2007; Nichols, 2016). Although the interaction with the 5-HT_2C_ receptor is thought to be involved to some extent in overall profile of psychological effects induced by psychedelics, the 5-HT_2A_ receptor is considered as the main primary target mediating the action of psychedelics in humans (Vollenweider et al., 1998; Nichols, 2004; Nichols, 2016; Kraehenmann et al., 2017; Preller et al., 2017).

The moderate selectivity for the 5-HT_2A_ receptor over 5-HT_1A_ and 5-HT_2C_ receptors observed for the 4-alkyloxy substituted derivatives (structures **6**–**8, 12**–**14, 18**–**19**) is in agreement with previously reported selectivity ratios for various substituted phenethylamines (Barfknecht and Nichols, 1975; Glennon et al., 1983; Pierce and Peroutka, 1989; Glennon et al., 1994; Nichols et al., 1994; Nelson et al., 1999; Rickli et al., 2015; Luethi et al., 2018). However, this selectivity for the 5-HT_2A_ receptor is not seen for tryptamine psychedelics or LSD, which are non-selective at these serotonergic receptors (Halberstadt and Geyer, 2011; Rickli et al., 2015; Luethi et al., 2018). Furthermore, a previous study shows that the 5-HT_2A/2C_ receptor binding of various psychedelics *in vitro* can be used to predict the clinical potency in humans (Luethi and Liechti, 2018).

### Binding to the 5-HT_1A_ Receptor

The 2C-O derivatives bound with low affinities to the 5-HT_1A_ receptor (*K*_i_ = 2700–5500 nM), similar to 2,4,5-trisubsituted *N*-2-methyoxybenzyl (NBOMe) derivatives (*K*_i_ = 1800–7100 nM) (Rickli et al., 2015) and slightly weaker than 2C-T derivatives (*K*_i_ = 660–2368 nM) (Luethi et al., 2018). Some 2C derivatives lacking 4-oxo or 4-thio substitution, such as the 4-bromo derivative 2C-B, and some psychoactive tryptamines displayed more noteworthy binding at the receptor in earlier studies (Rickli et al., 2015; Rickli et al., 2016) but did not reach the low nanomolar 5-HT_1A_ affinity of LSD (Luethi et al., 2018).

Structural modifications of the amphetamine derivatives **13**-**19** did not result in 5-HT_1A_ receptor binding in the examined concentration range for any of the compounds. Although the aryl-unsubstituted derivatives amphetamine and phenethylamine share little to no pharmacological properties with the psychedelic phenethylamines, it has been shown that amphetamine (*K*i = 6700 nM) (Simmler et al., 2013) has an affinity of more than one order of magnitude lower than phenethylamine at the 5-HT_1A_ receptor. This suggests an unfavorable role of the α-methyl group towards binding abilities at this receptor. This diminishment of binding affinities upon α-methyl introduction into phenethylamines is in accordance with several other aryl-substituted phenethylamines investigated (Rickli et al., 2019).

### Binding and Activation of the 5-HT_2A_ Receptor

Carbon chain length extension and/or oxidation of the 4-alkyloxy substituent increased the binding affinity at the 5-HT_2A_ receptor 5-fold (2C-O-16; **8**) and 17-fold (2C-O-3; **7**), compared to 2C-O-2 (**6**), the derivative bearing the shortest carbon chain. Similarly, the binding affinity was increased 2-fold for the difluorinated 2C-O-21.5 (**10**) and 4-fold for the trifluorinated 2C-O-22 (**11**) when compared to the monofluorinated derivative 2C-O-21 (**9**). However, **9** displayed the most potent 5-HT_2A_ receptor activation and the highest activation efficacy among all fluorinated 2C-Os. Surprisingly, activation potency of 2C-O-21.5 (**10**) was in the high nanomolar range, 50-fold and 6-fold higher than for 2C-O-21 (**9**) and 2C-O-22 (**11**), respectively. Likely, difluorination on **10** is less favorable for higher activation potency than monofluorination (2C-O-16; **8**) or trifluorination (2C-O-22; **11**).

Similar trends were observed for 3C-O derivatives. The extension and/or oxidation of the 4-alkyloxy substituent increased the binding affinity up to 9-fold for MALM (**14**) and 21-fold for MMALM (**13**) when compared to TMA-2 (**19**). Furthermore, the activation potency at the 5-HT_2A_ receptor was 66-fold and 127-fold increased for compounds **14** and MFEM (**15**), respectively, when compared to **19**. The binding affinity was slightly increased (2-fold and 4-fold) for the difluorinated MDFEM (**16**) and trifluorinated MTFEM (**17**), respectively, when compared to the monofluorinated **15**. Increasing number of fluoride substituents also increased the activation potency, resulting in a 2-fold increase for **16** and a 17-fold increase for **17** when compared to **15**.

Where available, direct comparison of the 2C-Os to their amphetamine counterparts revealed slightly higher 5-HT_2A_ receptor binding, higher activation, and lower efficacy for 4-alkoxy-substituted phenethylamine compounds (2C-O-16 *vs.* MALM and 2C-O-3 *vs.* MMALM). Similar observations were made for the fluorinated derivatives with the exception of receptor activation, which differed for 2C-O and 3C-O derivatives. Overall, the 4-alkoxy-substituted 2,5-dimethoxyphenethylamines activated the 5-HT_2A_ receptor as partial agonists (A_E_ = 30 – 84%), meanwhile the 4-alkoxy-substituted 2,5-dimethoxyamphetamine counterparts showed slightly higher activation efficacy (A_E_), with some amphetamine counterparts activating the 5-HT_2A_ receptor as full agonists (A_E_ > 85% –95%; compounds **13** and **14**). These results suggest that the α-methyl group plays a minor role in 5-HT_2A_ interactions for the tested compounds_._ This finding is in line with previous reports that the racemic α-Me introduction causes largely unchanged effects on the binding affinity and functional potency at the 5-HT_2A_ receptor affinity but does augment the intrinsic activity [Nichols et al., 1994; Parrish et al., 2005; Trachsel et al., 2013)].

Compounds 2C-O-1 (**21**) and 2C-O-4 (**22**), two members of the 2C-O family, were not psychoactive in humans, at least at the doses tested so far (Shulgin and Shulgin, 1991). It has been suggested that this may be due to a rapid metabolism or low binding affinity to the 5-HT_2A_ receptor (Clark et al., 1965; Nelson et al., 1999; Trachsel, 2012). The 5-HT_2A_ activation mediates psychedelic effects (Glennon et al., 1992; Chambers et al., 2002; Kraehenmann et al., 2017) and receptor binding affinity has been shown to be a good predictor of the dose needed (clinical potency) to induce a psychedelic effect (Luethi and Liechti, 2018).

The amphetamine derivatives and 2C-Os studied herein bound with moderate to high affinity to the 5-HT_2A_ receptor and are partial or full agonists at the 5-HT_2A_ receptor, rendering them potentially psychedelic. In previous studies, 2C-T and NBOMe derivatives were shown to bind to the 5-HT_2A_ receptor in the low nanomolar range, and therefore more potently than most 4-alkyloxy substituted derivatives of the current study. However, with a binding affinity of 6.3 µM (Rickli et al., 2015), mescaline (**3**) exemplifies that even low binding affinities may result in strong psychedelic effects, when a sufficiently high dose (> 200 mg) is ingested (Shulgin and Shulgin, 1991). Therefore, by sharing many typical structural features with known phenethylamine-type psychedelics (Shulgin and Shulgin, 1991; Trachsel et al., 2013), all compounds investigated in this study may potentially elicit strong psychedelic effects.

In the present study, 2C-O-27 (**12**) showed the highest affinity at the 5-HT_2A_ receptor (*K*_i_ = 8.1 nM). This is consistent with previous studies that suggest that bulky 4-substituents, such as 4-benzylthio, of phenethylamines result in high affinity 5-HT_2A_ binding and antagonistic behavior (Dowd et al., 2000; Luethi et al., 2018; Luethi et al., 2019). Measured by head-twitch response (HTR), Halberstadt et al. reported equipotent behavioral potency for TMA-2 (**19**) and two 4-homologated analogs (MEM; **24** and MPM; **26**) (Halberstadt et al., 2019). Furthermore, the authors demonstrated that the determined potency for the investigated psychedelics *in vivo* using HTR correlated highly (r = 0.98) with previously reported human potency data. Among HTR, drug discrimination (DD) is an extremely powerful tool and has been used for decades in order to compare psychedelic compounds in rats. Thus, the 4-alkyloxy substituted derivatives of the current study which are predicted to be potentially psychedelic in humans (based on their 5-HT_2A_ receptor interactions determined herein) should be further investigated using HTR and/or DD to characterize their potential psychedelic effects in human.

Overall, within the series of compounds investigated herein, the highest activation potencies at the 5-HT_2A_ receptor subtype were observed for 2C-O-3 (**7**) and MMALM (**13**) (EC_50_ = 0.5 nM and EC_50_ = 1.5 nM, respectively). Compared to the reference psychedelic 2C-B, both **7** and **13** activate the receptor in the same range, with **7** showing 4-fold higher activation potency than 2C-B.

### Activation of the 5-HT_2B_ Receptors

At the 5-HT_2B_ receptor, for the 2C-O derivatives, the effect of increasing carbon chain length and/or bulkiness showed mixed effects on activation potency. The activation efficacy however was between 2-fold to 10-fold lower for the derivatives with increasing carbon chain (2C-O-16; **8**, 2C-O-3; **7** and 2C-O-27; **12**) when compared to 2C-O-2 (**6**). For the derivatives with increasing number of fluorine substituents, the activation potency was increased 2-fold (2C-O-21.5; **10**) and 4-fold (2C-O-22; **11**) compared to the monofluorinated derivative 2C-O-21 (**9**).

For the amphetamine-based derivatives, the effect of increasing carbon chain length/bulkiness, showed increasing activation potency at the 5-HT_2B_ receptor, that was 2-fold (MIPM; **18**), 28-fold (MALM; **14**), and 9-fold (MMALM; **13**) higher when compared to TMA-2 (**19**). However, whereas the activation efficacy which was substantially decreased for **18** in comparison to **19**, both **14** and **13**, were full agonists (for **18**, see discussions in Section 4.1). The derivatives with varying fluorinations showed similar activation potency at the 5-HT_2B_ receptor. The activation efficacy was however reduced by −20% and −55% for the difluorinated and trifluorinated, MDFEM (**16**) and MTFEM (**17**), respectively, when compared to the monofluorinated MFEM (**22**).

Taken together, these findings indicate that regarding 5-HT_2B_ activation, the extension of the carbon chain has the strongest effect on the amphetamine-based derivatives, whereas fluorination has the strongest effect on the phenethylamine-based derivatives. Previously, it has been reported that 5-HT_2B_ receptor activation may play a role in the mechanism of action of some substituted amphetamine type stimulants and mediate adverse effects like endocardial fibrosis (Fitzgerald et al., 2000; Rothman et al., 2000; Doly et al., 2008). For this reason, we examined the 5-HT_2B_ receptor activity for the 2C-O and 3C-O derivatives to estimate their potential to cause such adverse effects. We observed full agonist activation efficacy for 2C-O-2 (**6**) and amphetamine-based derivatives (MALM; **14** and MMALM; **13**) which could potentially lead to similar drug induced adverse effects (Rickli et al., 2015) for regular users.

### Binding to the 5-HT_2C_ Receptors

At the 5-HT_2C_ receptor, the effect of structural modifications resulted in similar differences in 5-HT_2C_ receptor interactions as observed at the 5-HT_2A_ receptor. The 2C-O derivatives with increasing carbon chain length showed an increase in binding affinity by 5-fold (2C-O-16; **8**), 153-fold (2C-O-3; **7**), and 20-fold (2C-O-27; **12**) when compared to 2C-O-2 (**6**). All fluorinated 2C-O derivatives (compounds **9-11**) displayed comparable affinity at the 5-HT_2C_ receptor.

For 3C-O derivatives, the extension of the carbon chain length enhanced the receptor binding affinity of MALM (**14**) and MMALM (**13**) 6-fold and 18-fold, respectively, when compared to the shortest carbon chain containing TMA-2 (**19**) derivative; extension of the carbon chain by addition of a propyl group (**18**) reduced the receptor binding affinity 2-fold when compared to the TMA-2 (**19**). In the case of the varying number of fluorine substituents, the binding affinity was increased 2-fold and ∼5-fold for MDFEM (**16**) and MTFEM (**17**), respectively, when compared to monofluorinated MFEM (**15**).

Overall, highest affinity (*K*_i_ < 1000 nM) at the receptor was observed for 2C-O derivatives with extended carbon chain modifications (2C-O-16; **8**, 2C-O-3; **7** and 2C-O-27; **12**) and for their amphetamine-based counterparts (MALM; **14** and MMALM; **13**). Previous findings show high affinity binding for the 5-HT_2C_ receptor in the range of 4.6–640 nM for NBOMe and 2C-T derivatives (Rickli et al., 2015; Luethi et al., 2018).

### Non-Serotonergic Monoamine Receptor and Transporter Binding Interactions

In regards to binding at other monoamine receptors, only 2C-O-2 (**7**) and 2C-O-22 (**11**) activated the human TAAR1. However, activity at the human TAAR1 is known to be lower for many psychoactive substances compared to rodent TAAR1 (Simmler et al., 2016). At the rat TAAR1, the binding affinity was increased ∼2-fold (2C-O-16; **8**), 3-fold (2C-O-3; **7**), and 16-fold (2C-O-27; **12**) for derivatives with increasingly longer carbon chains when compared to 2C-O-2 (**6**). The same was observed at the mouse TAAR1 with 2-fold (**8**), 4-fold (**7**), and 6-fold (**12**) increase in binding affinity. The binding affinity was slightly increased (2-fold) for the difluorinated (2C-O-21.5; **10**) and trifluorinated (2C-O-22; **11**) 2C-O derivatives when compared to the monofluorinated derivative, 2C-O-21 (**9**). At the mouse TAAR1, only the trifluorinated derivative **11** bound (*K*_i__=_ 2500 nM) while the monofluorinated and difluorinated derivatives did not.

None of the investigated amphetamine derivatives interacted with the human TAAR1 but all compounds bound to the rat TAAR1. MALM (**14**) and MMALM (**13**) bound the receptor with 3- and 5-fold higher affinity, respectively, than observed for TMA-2 (**19**) and MIPM (**18**). The addition of mono, di- or trifluorine substituents for MFEM (**15**), MDFEM (**16**), and MTFEM (**17**), respectively, had little effect on the extent of affinity at the rat TAAR1. The interactions of the amphetamine-based derivatives at the rat TAAR1 were mostly similar to those observed for their 2C-O counterparts. However, the increasing length of the carbon chain for 2C-O derivatives increased the extent of affinity at the receptor, which was not observed for the amphetamine-based derivatives. The extension of the carbon chain length for **14** and **13** allowed these derivatives to bind to the mouse TAAR1. Compounds **19** and **18** did not bind to the receptor in the examined concentration range. Similarly, only the tri-fluorinated derivative **17** bound to the receptor while the mono- and difluorinated derivatives **15** and **16** did not. A similar observation was made for fluorinated 2C-O derivatives, for which only the trifluorinated 2C-O-22 (**11**) bound to mouse TAAR1 in the investigated concentration range.

The rank order of affinity observed in our study for all the 2C-O and amphetamine-based derivatives at TAAR1 (rat > mouse > human TAAR1) was consistent with previous studies that investigated substituted phenethylamines with various bulky modifications (Lewin et al., 2008; Luethi et al., 2018; Luethi et al., 2019).

The 2C-O derivatives interacted with adrenergic α_2A_ receptors but there was no relevant binding to adrenergic α_1A_ and dopamine D_2_ receptors or any of the monoamine transporters. Binding selectivity for substituted phenethylamines for the α_2A_ over α_1A_ receptor is in support of previously published studies of 2C-T but not NBOMe derivatives, which have been shown to bind to both adrenoceptor subtypes (Rickli et al., 2015; Luethi et al., 2018). Additionally, the lack of binding to the monoamine uptake transporters observed for both phenethylamine-based and amphetamine-based derivatives is in line with previous studies of 2C derivatives which did not display significant affinity at monoamine transporters (Rickli et al., 2015; Luethi et al., 2018). An exception to this is 2C-O-27 (**12**), which bound to the DAT at 6.1 µM. Moderate affinity at monoamine transporters has recently been demonstrated for a series of 4-aryl substituted 2,5-dimethoxy phenethylamines (2C-BI derivatives) (Luethi et al., 2019). Compound **12** carries a phenyl ring in its 4-substituent as well and this feature therefore seems to increase transporter binding and potentially inhibition of 2C derivatives.

### Conclusion

In summary, we investigated the monoamine receptor and transporter binding and activation properties of several 4-alkyloxy-2,5-dimethoxy substituted phenethylamine and amphetamine derivatives *in vitro*. The compounds mainly interacted with serotonergic receptors and bound with the highest affinity to the 5-HT_2A_ receptor. This suggests that some of these amphetamine-based and phenethylamine-based derivatives could be potent psychedelics in humans.

The most active compounds with highest affinities, activation potencies, and activation efficacies were the 4-allyl and 4-methallyl derivatives 2C-O-16 (**8**) and MALM (**14**) as well as 2C-O-3 (**7**) and MMALM (**13**), respectively. Alterations of the 4-alkoxy group or introduction of fluorine substituents resulted in altered binding affinity at 5-HT_2A_ and 5-HT_2C_ receptors. Their low subtype selectivity is in line with the many other phenethylamine pharmacophore ligands tested so far. Nonetheless, subtle changes in chemical structure went in hand with changes in receptor profiles – and most probably in pharmacodynamics/pharmacokinetics – and would therefore likely lead to different types of psychedelic activities.

## Data Availability Statement

All datasets generated for this study are included in the article/**Supplementary Material**.

## Author Contributions

KK, DL, DT, and ML designed the research. KK and MH performed the research. KK and M.L analyzed the data. KK, DT, and ML wrote the manuscript with significant input from all other authors.

## Funding

This work was supported by the Federal Office of Public Health (grant no. 16.921318). DL was supported by a postdoctoral fellowship from the Swiss National Science Foundation (grant no. P2BSP3_181809).

## Conflict of Interest

DT is an employee of ReseaChem GmbH and MH is an employee of F. Hoffmann-La Roche.

The reviewer SB declared a past co-authorship with some of the authors DL, ML to the handling editor.

The remaining authors declare that the research was conducted in the absence of any commercial or financial relationships that could be construed as a potential conflict of interest.

## Abbreviations

2,4,5-trimethoxyamphetamine, TMA-2; 2,4,5-trimethoxyphenethylamine, 2C-O-1; 2,5-dimethoxy-4-(2-fluoroethoxy)amphetamine, MFEM; 2,5-dimethoxy-4-(2-fluoroethoxy)phenethylamine, 2C-O-21; 2,5-dimethoxy-4-(2,2,2-trifluoroethoxy)amphetamine, MTFEM; 2,5-dimethoxy-4-(2,2,2-trifluoroethoxy)phenethylamine, 2C-O-22; 2,5-dimethoxy-4-ethoxyamphetamine, MEM; 2,5-dimethoxy-4-ethylamphetamine, DOET; 2,5-dimethoxy-4-isopropoxyamphetamine, MIPM; 2,5-dimethoxy-4-isopropoxyphenethylamine, 2C-O-4; 2,5-dimethoxy-4-methallyloxyamphetamine, MMALM; 2,5-dimethoxy-4-methallyloxyphenethylamine, 2C-O-3; 2,5-dimethoxy-4-methylamphetamine, DOM; 2,5-dimethoxy-4-methylphenethylamine, 2C-D; 2,5-dimethoxy-4-propoxyampehtamine, MPM; 2,5-dimethoxy-4-ethoxyphenethylamine, 2C-O-2; 3,4,5-trimethoxyamphetamine, TMA; 3,4,5-trimethoxyphenethylamine, mescaline; 4-(2,2-difluoroethoxy)-2,5-dimethoxyamphetamine, MDFEM; 4-(2,2-difluoroethoxy)-2,5-dimethoxyphenethylamine, 2C-O-21.5; 4-alkoxy-2,5-dimethoxyamphetamine, 3C-O derivatives; 4-alkoxy-2,5-dimethoxyphenethylamines, 2C-O derivatives; 4-allyloxy-2,5-dimethoxyamphetamine, MALM; 4-allyloxy-2,5-dimethoxyphenethylamine, 2C-O-16; 4-benzyloxy-2,5-dimethoxyphenethylamine, 2C-O-27; 4-bromo-2,5-dimethoxyamphetamine, DOB; 4-bromo-2,5-dimethoxyphenethylamine, 2C-B; 4-butoxy-2,5-dimethoxyamphetamine, MBM; 5-hydroxytryptamine (serotonin), 5-HT; dopamine transporter, DAT; dopamine, DA; norepinephrine transporter, NET; norepinephrine, NE; serotonin transporter, SERT; trace amine-associated receptor 1, TAAR1.

## Supplementary Material

Supplementary figure 1 Monoamine transporter inhibition. Compounds were tested at a single high concentration (10 µm). Data are mean and SEM of three experiments. None of the compounds with the exception of MDMA relevantly reduced monoamine uptake (all EC_50_ values > 10 µM). As expected, the positive control MDMA markedly reduced NE and 5-HT uptake and moderately reduced DA uptake into the cells at 10 µM indicating activity at all transporters. The moderate inhibition of the DAT is due to the known low potency of MDMA at this transporter in this assay (EC_50_ = 17 µM).
