# Supplementary figures and images for: Receptor Interaction Profiles of 4-Alkoxy-Substituted 2,5-Dimethoxyphenethylamines and Related Amphetamines
Source: Front Pharmacol. 2019 Nov 28;10:1423. doi: 10.3389/fphar.2019.01423 (PMC6893898; doi:10.3389/fphar.2019.01423)

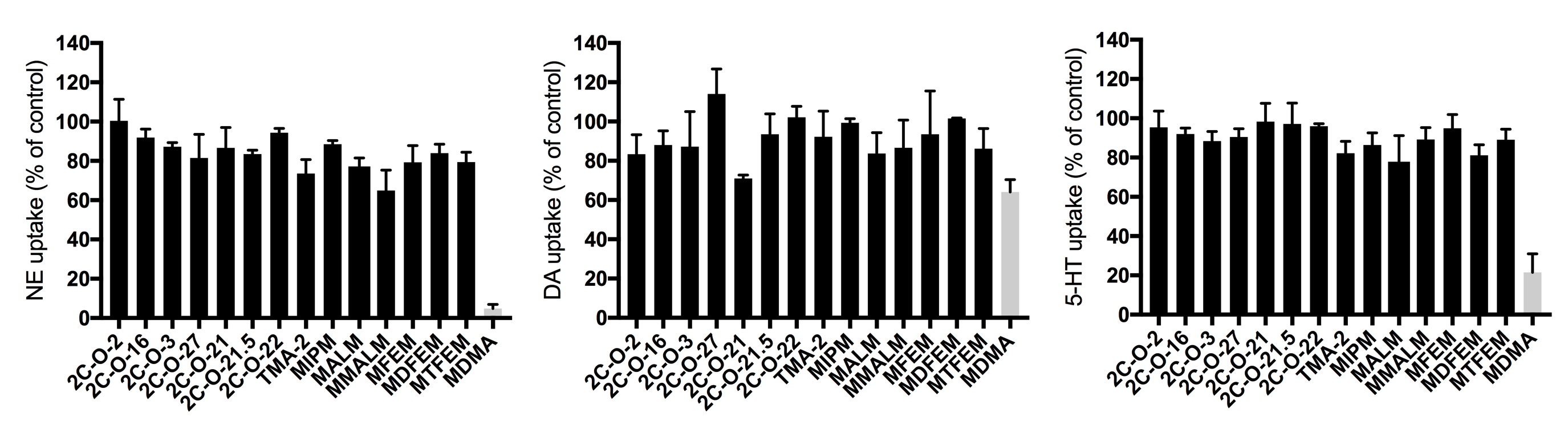

Supplement: Supplementary figure 1 — Monoamine transporter inhibition. Compounds were tested at a single high concentration (10 µm). Data are mean and SEM of three experiments. None of the compounds with the exception of MDMA relevantly reduced monoamine uptake (all EC50 values > 10 µM). As expected, the positive control MDMA markedly reduced NE and 5-HT uptake and moderately reduced DA uptake into the cells at 10 µM indicating activity at all transporters. The moderate inhibition of the DAT is due to the known low potency of MDMA at this transporter in this assay (EC50 = 17 µM). [file Image_1.tiff]
